# ‘Other’ Posts in ‘Other’ Places: Poland through a Postcolonial Lens?

**DOI:** 10.1177/0038038514556796

**Published:** 2016-02

**Authors:** Lucy Mayblin, Aneta Piekut, Gill Valentine

**Affiliations:** University of Sheffield, UK; University of Sheffield, UK; University of Sheffield, UK

**Keywords:** diversity, Eastern Europe, modernity, Poland, postcolonialism, Warsaw

## Abstract

Postcolonial theory has tended to focus on those spaces where European colonialism has had a territorial and political history. This is unsurprising, as much of the world is in this sense ‘postcolonial’. But not all of it. This article focuses on Poland, often theorised as peripheral to ‘old Europe’, and explores the application of postcolonial analyses to this ‘other’ place. The article draws upon reflections arising from a study of responses to ethnic diversity in Warsaw, Poland. In doing so we conclude that postcolonialism does indeed offer some important insights into understanding Polish attitudes to other nationalities, and yet more work also needs to be done to make the theoretical bridge. In the case of Poland we propose the ‘triple relation’ be the starting point for such work.

## Introduction

Postcolonial theory has tended to focus on those spaces where European colonialism has had a territorial and political history. This is unsurprising, as much of the world is in this sense ‘postcolonial’. But what of those postsocialist states to the west of the ‘East’ and the east of the ‘West’? Former Soviet ‘colonies’ experiencing new western imperialisms at the same time as adjusting to their ‘transition’ to capitalism? Postcolonial theory has much to offer to social and cultural studies of postsocialist spaces and a growing number of scholars in Eastern Europe have been arguing as much in recent years, particularly in Poland (Carey and Raciborski, 2004; Cavanagh, 2004; Deltecheva, 1998; Janion, 2006; Kania, 2009; Kuus, 2004; Owczarzak, 2009; Pickles, 2005; Skórczewski, 2006, 2009; Todorova, 1997; Zarycki, 2011). Yet there are limitations in this literature, particularly among those who offer a ‘comparative empires’ reading of postcolonial and postsocialist spaces. In this article we offer a reading of everyday understandings of diversity in Poland using postcolonial theory. Our intervention is crucially to argue that contemporary ideas of Polishness and otherness might be understood in terms of a *triple relation*: Poland as former colony, as former coloniser and finally in relation to the western hegemons.

In Poland, the experience of socialism and the aftermath of 1989 are fundamental to understanding political and public experiences and understandings of difference and diversity in the country (Kania, 2009). And yet, while the ‘postsocialist condition’ (Stenning, 2005) is important for understanding the nation and its response to difference, this is not the only lens through which one might look. A more long-term perspective, and a more complex vision of Polish society which reaches beyond postsocialism as the focus for analysis, can offer new insights. Presocialist histories are important in thinking through contemporary articulations of Polish national identity, particularly in terms of Polish dominance over others in the near East. The history of Poland is also cut through with ‘colonialisms’ – Poland experienced Soviet imperialism and was itself an imperial power in the Eastern European region. More recently, Poland has turned westwards and sought to ‘return’ to Europe (both politically and culturally) and in a sense to learn to be European again, for example through the European Union (EU) and NATO enlargement processes (Kuus, 2004). The old colonial powers of Western Europe, within this context, have exerted significant imperial influence over trajectories of social difference in multiple spheres of national life.

We draw here on data from in-depth biographical interviews with Poles living in Warsaw conducted within a larger research project ‘Living with difference in Europe: Making communities out of strangers in an era of super mobility and super diversity’ (see Mayblin et al., 2014; Piekut et al., 2012; Valentine et al., 2014). On the basis of a representative survey on attitudes and encounters with difference in Warsaw (*N* = 1499), 30 participants were selected for a qualitative study. Three interviews were then conducted with each participant in Polish over a one year period in 2012 (*n* = 90). Each interview explored different ‘scales’ of experience with difference: individual, approached as a life history interview; urban, discussing changes in Warsaw in terms of diversity; and national, investigating general views on relations between Poles and various minorities. The research participants represented a range of demographic characteristics, in terms of age, (dis)ability and socio-economic status, with some representatives of minority sexual, religious and ethnic groups (see summary of respondents’ profile in Table 1). Interviews were verbatim transcribed, coded and analysed using qualitative data software. We draw on these data to illustrate the means by which postcolonial theories and concepts might offer insight into research in Poland today. More specifically, we propose that thinking Poland postcolonially offers much in terms of understanding both national identity and ideas of ‘otherness’ in the country.

**Table 1.** Demographic profile of respondents (*N* = 30).

Characteristic		No.	Characteristic		No.
Gender	Female	15	Nationality	Polish	28
	Male	15		Other	2
Age group	18–34	11	Religion	Catholic	25
	35–59	12		Other religion	2
	60+	7		No religion	3
Marital status	Single	13	Place of birth	Warsaw	13
	Married	11		Other city in Poland	16
	Other	6		Abroad	1
Disability	No	25	Work status	Student	6
	Yes	5		Employed	18
Sexual orientation	Heterosexual	28		Unemployed	2
	Other	2		Retired & permanently sick	4

## Poland through a Postcolonial Lens

In recent years a growing number of scholars in Poland have begun to explore the possible application of postcolonial concepts (see Janion, 2006; Kania, 2009; Skórczewski, 2006, 2009; Zarycki, 2005), predominantly drawing on an analysis of discourse. This work falls into two divergent strands which might be labelled ‘comparative empires’ and ‘theoretical insights’. Such discussions rarely draw on individual narratives which reflect everyday encounters with difference. In the comparative empires perspective Poland is seen as a country historically colonised by Soviet Russia. The contemporary situation can therefore be interpreted in the same way that the postcolonial experience of other European colonies might be understood. Here, the central questions are around Polish identity (and anxieties around identity) in relation to their former Russian overlords (Fiut, 2007: 34). This line of investigation has clear limitations, not least in the practical complexity of Soviet colonialism, the question of whether the ambition of world socialism ‘counts’ as colonialism, and the local articulations of the relationship. Furthermore, this approach also falls victim to the central danger of this intellectual project: re-inscribing the colonial relation between East and West. Furthermore, postcolonial theories emerged in connection with leftist discourse and were mainly developed by Marxist scholars during the Cold War. This, as Korek (2007) has pointed out, makes the notion of Soviet Russia being a colonising power problematic. Soviet Russia supported the decolonisation process of countries that were ‘oppressed by capitalism’, taking the role of the only non-colonial empire.

The promise of postcolonial theory is not, we would argue, in engaging in the work of comparative empires, or to say that the postsocialist East can be subsumed into a postcolonial understanding of the world which foregrounds the western empires. Rather, where the application may work is through using some of the tools of postcolonial theory to better understand the Eastern European experience, while also acknowledging that the hegemonic discourse of western enlightenment has a variety of spheres of influence, one of which is within Europe itself. Some postcolonial concepts might therefore be helpful here, such as: orientalism, hybridity, giving voice and speaking back, time as space and contesting the project of ‘modernity’ (from modernisation to multiple modernities and beyond) (Bhabha, 2005 [1994]; Bhambra, 2007; Eisenstadt, 2002; Said, 1978, 1994; Spivak, 1988).

Postcolonialists, over the past 35 years, have called for a dramatic change in the way colonialism is approached. The central concern is with the narrative of modernity. Modernity has both temporal and geographical dimensions. The temporal concerns rupture – the idea that at some point in time something happened to western societies which transformed them from pre-modern into modern societies. The Renaissance, the French Revolution and the industrial revolution form the key pillars of this story, together facilitating the Enlightenment, the emergence of democracy and the rise of capitalism in the West (Bhambra, 2007). This narrative reaffirms the idea that some places in the world are today modern, while some are not. Combined with the temporal variable, this logically means that some societies are ‘behind’ western societies, existing in their past rather than in a global present. Modernity is therefore commonly theorised as simultaneously distinctive and Western European in its origins. What is interesting for our case is that postcolonial scholars often generalise about Europe, implying that the whole continent might be subsumed into their critique. And yet not all of Europe pursued representative democracy, capitalism or human rights (key indicators of modernity) at the same point in time as the ‘western core’. This peripherality to conceptions of modernity raises interesting questions for sociology in postsocialist spaces.

In looking at attitudes towards Poland and Poles in Western European countries through a postcolonial lens one can observe politicians, the media and the public at large drawing on colonial tropes of East and West, setting Poland within a wider civilisational hierarchy (Spigelman, 2013). However, if we look at perspectives from within Poland then the discourses drawn upon are different – unsurprisingly, the relation is not reversed. There is, in fact, a triple relation apparent: the relation to Russia (complex in itself as this was not an example simply of another colonialism), and then there is a countervailing relation to ‘the West’ as an alternative ideological hegemon, the discourse around which draws on themes of western superiority, on orientalism. Then, there is the relation to eastern and third world ‘others’, including those living in the pre-war Polish territories in the near East, who are often viewed in civilisational terms. Poland’s position within this discursive framing is not simply an ‘inbetweeness’ (in between East and West), as some scholars have argued (Galbraith, 2004; Janion, 2011), it is something much more complex. These three axes operate in parallel, and the outcomes of competing discourses, spheres of influence, racial and social hierarchies, distinctions between ‘insider’ and ‘outsider’, the ‘self’ and the ‘other’ manifest themselves in complex and contradictory ways.

This triple perspective resonates with Kuus’s (2004) analysis of the European discourse on EU and NATO enlargement. Kuus suggests that the Cold War era binary division of Europe into communist and capitalist changed in the early 2000s, as powerful European actors began to divide the continent into three different regions: the European/Western core, the Central European applicants and eastern peripheral states which are not yet European enough to join the EU (e.g. post-Soviet republics), or at all (i.e. Russia, Turkey). Kuus (2004) has proposed that other studies should look at how these ‘othering’ frames are used in the ‘power margins’ (Central and Eastern European countries). This article follows this question by situating ‘inscriptions of otherness’ in the Polish historical and geo-political context. Specifically, we explore how the triple relation influences people’s responses to diversity and how the responses are aligned with different narratives of modernity. The next three subsections address the three aspects of the triple relation.

### Poland as a Formerly Colonised Country

Poland has experienced multiple histories of colonisation by external powers. In the 18th century Poland disappeared from the European map and the country was partitioned three times – by the Russian Empire, the Kingdom of Prussia and the Austrian Monarchy (1772, 1793 and 1795). Poland was deprived of sovereignty for 123 years, during which time ethnic Poles were pressured into cultural assimilation and experienced discrimination as a minority. Partial sovereignty was regained after the Congress of Vienna in 1815 which resulted in the creation of the Kingdom of Poland (1815–1918, known also as Congress Poland or ‘Vistula Land’ (rus.: *Priwislinskij Kraj*)). Poland remained politically integrated with Russia, though seized some limited independence after failed uprisings in November 1830 and January 1863. From 1945 to 1989 the Polish People’s Republic (PPR) was a satellite of Soviet Russia. This period has been recognised by many as another colonisation, with Soviet Russia acting as a coloniser (Moore, 2001) or as a semi-coloniser (Carey and Raciborski, 2004), since Poland was officially an independent state, but its internal and international politics were profoundly controlled by the leaders of the Soviet bloc countries.

As this brief historical account shows, Poland has experienced multiple phases of colonial domination in a variety of forms. This, and particularly the 20th-century experience of independence and independence-in-domination by Russia, has had a profound impact upon contemporary Polish national identity (Janion, 2011). Some scholars argue that a dislike of the Russian people is the ‘glue that holds Polish identity together’ (Janion, 2011: 6). One of the central popular anxieties around relations with Russia is the perception of Russia as a threat. The stereotype of ‘threatening Russia’ was reinforced during the Second World War and communicated to younger generations. One of our respondents, a woman born in the inter-war period in formerly Polish Vilnius, shared painful stories about the wartime period and post-war resettlement in Poland. When referring to Russian people or language she always used the disrespectful term ‘*Ruski*’ (so called ‘Russkis’). Her father fought in the Home Army, which did not accept the pro-Soviet communist authorities that emerged at the end of the war. Thus, as she explains, the hatred of Russia ‘came from home’. While her husband was a Communist Party member sympathising with the Soviet Union, her son was virulently anti-Russian. When her son refused to learn Russian at school she felt ambivalent:

My [son] said, for example, he will not learn *Ruski* in school. I went to my son’s [school] (…), I was constantly called by *Ruski* teacher to come there, [because] he won’t learn *Ruski*. [My son said] ‘I won’t learn!’ [*Happily*] I admired him on the one hand, he is so tough, I was delighted, but I had straight A’s in *Ruski* (…) I guess, because I was brought up there, in those regions and I was quite good at *Ruski*. I was good. I still buy *Ruski* [cigarettes], I read to my grandson what is written here [Respondent shows cigarettes with Russian inscriptions], (because) they are cheaper. (Alina, 76)

Another woman reflected on her memories about Russian people who lived in Warsaw during the socialist period. She perceived Russian people as those who ‘stifled’ Poles. Today, though, she is aware of her prejudice and is self-critical, yet she cannot escape it:

As I tried to get a job, I went to the Ministry. One day I was standing at the door, waiting for someone to come (…) [and] I heard a conversation in Russian on the phone. And my hair stood on end. What are the Russians doing in our Ministry of Education? I’ve always been so suspicious about them. When I heard the conversation in Russian on the street I thought ‘Oh, those people again, those who want to stifle us here.’ At the moment I no longer have this very suspicious and reluctant attitude to them, but I can’t say I love them. I am aware that authority and society are two different things, but society is unfortunately prone to do what authority says. (…)

Did you meet any Russians here in Warsaw?

No, I didn’t meet any Russians, and probably wouldn’t want to. I’d be afraid that I could at some point show my dislike and someone would be sorry. (Danuta, 67)

In this account the respondent admits that Russia no longer poses any threat to Poles and yet fears around being stifled, as she puts it, continue. Following 1989 the popular press has framed the Russian threat as an issue of gas supply, and more recently in the form of conspiracy theories around the Smoleńsk presidential plane crash in 2010, which happened close to one of the symbols of martyrdom of Poles during the Second World War (Zubrzycki, 2011).

In countering this sense of threat, Poles have developed multiple negative representations of Russians which cast Russia as weaker politically, more ‘backward’, and less civilised – the former ‘coloniser’ has become ‘the other’. For example, in discussing the debate on the possibility of introducing same-sex civil partnership in Poland, one respondent used Russia as a reference society whose fate Poles should avoid. In this context, Russia remains ‘less advanced’ from the perspective of the European narrative on modernity in that it lacks compliance with international equality laws. Poland possesses a moral superiority and could avoid the Russian fate (Zarycki, 2004). Therefore, rather than depicting Russian equality and human rights laws as simply ineffective or limited, the discourse sites this limitation within a temporal and spatial narrative – some countries are lagging behind the modern West and should catch up. Lagging behind is implicated in lacking ‘civilisation’.

This orientalising perspective has been transposed onto Polish regions that were governed by Russia during partition. The post-Prussian and post-Austro-Hungarian regions are remembered as regions of prosperity and modernisation, while any economic successes and progressive social and voluntary work that occurred in the past and are occurring today in the formerly Russian regions are silenced or forgotten (Zarycki, 2008). This ‘discourse of competences’ is often applied to the eastern territories of Poland, which are perceived as more ‘backward’ because of their historical connections with Russia, while the positive legacies of Russian influence in these regions are overlooked (Zarycki, 2011). Negative views on these regions especially prevail among young people. For example, one interviewee described the town where his grandparents live in eastern Poland as a more superstitious place and with amusement, mimicking the eastern Polish accent.

The social and cultural hegemony of the Russian Empire was therefore only marginally successful in Poland. Some scholars link this failure of the Russian colonial project with the Polish ‘inferiority–superiority complex’ – a sense of inferiority in relation to the West, alongside high levels of Polish national pride (Kurczewska, 2003). Perhaps because Poles feel inferior (insufficiently modern and European) in relation to the West (Kuus, 2004), they have developed a disrespectful attitude towards other ‘more eastern’ and ‘even less European’ countries. However, in the Polish context the negotiations of ‘easternness’ are marked by centuries of difficult history of being neighbours or citizens within one national organism; a history that spans well beyond the recent post-1989 transformations and EU enlargement.

### Poland as a Coloniser: ‘Orientalisation’ of Borderlands

The territory of present day Poland, like many states, differs considerably from previous incarnations of the country. From the 14th century up to 1945 the Polish eastern borders were located approximately 200 km south-east of their present location, incorporating the territories of contemporary western Belarus, western Ukraine and eastern Latvia. Following considerable changes to the Polish territories introduced with the Yalta Conference in 1945, Poland was moved westwards, and the eastern territories were lost while some western and northern regions (including almost the entire Upper and Lower Silesia, Pomerania, Lubusz Land, parts of Greater Poland, Kuyavia, Warmia and Masuria) were incorporated and named the Regained Territories. Polish people living in the former Polish territories in eastern neighbouring countries were repatriated to Poland and mostly to the Regained Territories in parallel with German repatriations in the same area.

The lost eastern territories have colloquially been named the ‘Eastern Borderlands’ and over time a nostalgic and idealising discourse built upon mythologies of a ‘lost homeland’ created during the partitions has emerged (Bakuła, 2007). However, historically these eastern territories were never considered to be ethnically Polish in terms of their population. The territories of contemporary Belarus and Ukraine were conquered in the 14th and 15th century by the Kingdom of Poland and the Grand Duchy of Lithuania and incorporated into the Polish–Lithuanian Commonwealth (common elective monarchy, 1569–1795). These territories were a mix of people of different ethnicities and religions (Catholic, Orthodox and Jewish). People in the Commonwealth had, in theory, the same rights and privileges irrespective of their ethnicity (introduced by the Warsaw Confederation 1573), but the rights were limited to nobility (10 per cent of the population). Ethnic and religious differences were interwoven with social and class divisions, with Polish gentry, who had more economic resources and power, being significantly advantaged in this context (Gella, 1989: 13; Snochowska-Gonzales, 2012).

During the Commonwealth period the eastern border was perceived by Poles as a territory where nomadic Cossacks, Tartars and people who came as ‘fugitives from serfdom’ led ungoverned lives, so it was represented both as a space of freedom and a space of fear (Janion, 2006). These perceptions reflect one of the central national myths – Catholic Poland as a bulwark of Christendom defending Europe against the infidel, against the barbarian and against the Asian threat (Wise, 2010a; Zubrzycki, 2011). The Borderlands became a space of nationalistic tension in the time of Poland’s regaining of independence, specifically with Ukrainian people (e.g. war over the Eastern Galicia regions, 1918–1919, ‘Massacres in Volynia’, 1943–1944). The imperialistic approach towards people living in these territories (assimilation was expected into the socio-cultural norms of contemporary Poland) and the homogenising mythologies of the Polish national identity were strengthened by socialist national policy (Copsey, 2008). All these experiences have been incorporated into the national collective memory (Konieczna, 2001). One of our research participants was born in south-east Poland in an area which until 1945 was Polish and is currently Ukrainian, a region of Polish–Ukrainian clashes in the period 1943–1944. Stories of the massacre were passed to younger generations and the term ‘Ukrainian’ has become a powerful symbol of the ‘other’ that has deeply shaped her attitudes, even to things that belong to the Ukrainian people, such as animals: ‘it’s not our dog, but Ukrainian dog, we can say, dog which belongs to people who murdered us. Somehow, it’s a psyche, it’s scary’ (Urszula, 52). When she moved to Warsaw she rented a room in her flat to a girl who dated a Ukrainian boy. The couple broke up and in her justifications she drew a connection between her beliefs regarding Ukrainians and her encounter with the Ukrainian boy:

I had some terrible expectations of Ukrainians, really terrible. They told me that they were there, they killed, they murdered, they nailed children to walls, it was simply, it’s what I heard from my grandma, my grandma told me such stories (…)

And in the course of time, when you got to know this boy better, did your attitude change? How was it?

I mean, that’s why I imagined that, that he may hurt this girl. Somehow, I didn’t feel it would be all right. And it turned out that he hurt her because he left her afterwards. He left her. He promised wonders, he promised to marry her and so on, that they would be together, and then he said: ‘You know, I want to date also other women.’ (…) she slept with him. Even, I shouldn’t say it, but he perhaps infected her, because she visited a gynaecologist and she said that she contracted some disease (…). So I don’t think of Ukrainians in positive terms, quite the contrary, my attitude is, that they are, we shouldn’t generalise, maybe it’s only my imagination, maybe I don’t know, maybe somebody says it and I just repeat it. (Urszula, 52)

These memories around the brutality of Borderland relations have been interpreted as indications of failings in the Ukrainian character. The Ukrainian boy is racialised, since his actions are not seen as personal failings, but as a reflection of his ‘Ukrainian nature’. The contemporary migration of Ukrainians, Belarussians and citizens of other eastern neighbour countries (the major immigration source) adds another dimension to discursive constructions around the relations with the ‘Borderlands’. ‘Borderland people’ are again living among Poles, and more importantly, many of them have some Polish roots or family relations (Konieczna-Sałamatin, 2011), so the association of these people with ‘otherness’ becomes complicated by the banal realities of everyday life. A common representation of Ukrainians as a poor and economically ‘backward’ nation, and the association of Ukrainian immigrants with undocumented migration and illegal working (Konieczna, 2001) was also present among our respondents. One respondent in our research spoke of her Ukrainian sister-in-law. In her story she relates her son’s immigration experience, who lived in the UK and now resides in Sweden, to her own experiences in the labour market. Working as a domestic help she competes with mostly Ukrainian women as she says, ‘I don’t mind that she is Ukrainian at all’:

You know what, but if our [Polish] people move to other countries, then they perceive us similarly there. (…) So this is the natural order, everybody takes away [jobs]. Sure, I was annoyed many times because they [Ukrainian women] raise price, they unreasonably raise prices. (…) Well, because there are a lot of them, for example cleaning and keeping a house. There are a lot of these Belorussians and Ukrainians and they take up jobs and raise prices, they even have higher salaries than we do. (…) So, yeah, something has changed. Back then, I didn’t mind, now it is a bit different. (…) And it is because they are employed more often … Because we have got families, we come back somewhere. And they usually stay over [in Poland]. (…) Possibly, they come and are ready to work at anyone’s beck and call. Unlike us. Because I’ve got eight hours and I go back home. So it is different. Well, but for example my sister-in-law works shorter hours in Poland and has the same salary as I do. (Celina, 58)

Similar stories were shared by other respondents who perceived economic immigrants from the eastern neighbouring states as those who do not ‘deserve’ to have the same salary as Polish people. However, through the same means of migratory experience Poland has moved closer to the ‘western core’, because it has become an attractive destination country for immigrants. These accounts provide evidence that citizens of the Central and East European countries negotiate their own degrees of ‘easternness’ or ‘westernness’ in relation to other countries of the region (Kuus, 2004), especially those in close proximity (Siemieńska, 1996). Past Polish emigration was compared with contemporary immigration, particularly from the eastern neighbouring countries:

Although their [immigrants] status is certainly worse, like usually in the case of *gastarbeiters* [ger.], but … it tickles my national pride, that we used to go to Germany to pick up strawberries and we went to *saksy* [pol., a colloquial term for a seasonal job abroad], to a *bauer* [ger.]. (…) And now we’ve become ‘the West’ and other nations come to us, and we are almost these masters [pol. ‘paniska’]. We give them jobs, they clean up, they build, they babysit. (Henryk, 66)

In this relation ‘modernity’ appears as a ‘colonisation of space and time’ (Mignolo, 2011: 6); this ‘lower’, less empowered social positioning is ascribed to a specific region (Eastern Europe), but is also associated with the ‘past’ Poland, and thus is presented as more immature. The narrative on the Borderlands is also reflected in the feeling of responsibility, emotional attachments (‘lost homeland’) and, in turn, a paternalistic approach towards these regions which could be taught by Poland how to become European (especially Ukraine, Bakuła, 2007). Reverse mechanisms seem to be in operation in relation to Jewish people, who also represent a former multicultural facet of pre-war Poland, but are not visible and encountered on the same daily basis as immigrants from the East are. This group was ‘imagined’ by respondents as more dominant in relation to Poles (understood in socio-economic terms and power relations; see Kofta and Bilewicz, 2011), which could not be ‘orientalised’ and described as a ‘backward other’ (Snochowska-Gonzales, 2012). It could be argued that this ‘colonising incapacity’ constitutes a source of uneasiness and prejudice. As such, the triple relation does not fully explain anti-Semitism or any other prejudice, but it helps to uncover how everyday encounters with ‘others’, even if they are ‘imagined encounters’, are relationally bound with different narratives of hegemonic relations and contestations of previously ‘subaltern’ positioning of Polish people.

### Poland and the Western ‘Hegemons’

Until the 9th century Poland was a pagan country. In the 10th century the Polish duke Mieszko I decided to convert to Christianity, but according to Latin, as opposed to Slavonic, rite. Since then Poland has had a stronger relation with western Latin religious culture and thought. Janion (2006) sees this event in Polish history as the starting point of a national identity split between the East (represented by Slavdom) and the West. The subsequent history of Poland, during which stronger links with the Russian Empire developed, has only reinforced this tension. Some scholars argue that the aspirations of being included into Western Europe and accepted as not a ‘barbarian Slavonic’ people, has led to the creation of a para-colonial relationship with western countries (Buchowski, 2006; Kuus, 2004; Thompson, 2010).

Thompson (2010) describes the relationship between Poland and the ‘West’ as a ‘surrogate hegemon’. She traces its roots in the period of partitions (1773/1795–1914) and argues that similar processes were at work in the socialist period. In both periods large numbers of Polish intelligentsia emigrated from Poland and with them the narrative on Polish socio-cultural life was relocated outside Poland. The narratives developed by Polish intellectuals in Western Europe – who were seeking explanations for the partitions or commented on internal affairs in socialist Poland – confirmed the inferiority of Polish society and, according to Thompson (2010: 4; for critique see Snochowska-Gonzales, 2012), Poles started internalising the orientalising gaze of the West, but at the same time ‘they tended to transfer the notion of inferiority onto the lower social strata in Poland, or onto those strata that did not subscribe to the Enlightenment slogans about progress and secular development of humanity’. Through the decades Poles adopted the discourse of the conquerors, blaming themselves for the failure of the Polish state, at the same time as the belief in western supremacy grew stronger. This orientalist perspective which casts Poland as traditional and behind the West was present among our informants too. Reflecting on changes that have occurred in Poland in the last two decades, Jakub perceives current public debates in Poland, those represented mainly by politicians who shape the discourse, as parochial in relation to western political culture. He explains:

When you are saying that Poland is parochial, then?

(…) But still in certain situations, being parochial means that we are far away from this Western Europe, we are far away in terms of thinking, perceiving certain issues. And, as I’ve mentioned, politicians are to blame. (…)

What’s the difference between thinking of western politicians and our Polish politicians?

I will put it differently – our politicians’ mentality in certain cases, as I’ve said, with respect to otherness, generally, otherness, and so on, they are simply, they think like in the past. (Jakub, 36)

This description of ‘thinking like in the past’, while western politicians presumably think in a new mode which is distinct from old ways of thinking, reflects the articulation of ideas of modernisation and progress which are often taken for granted. The year 1989 brought independence and a chance to re-establish and reassess relationships with both the West and the East. Neoliberal politics privileged the modernisation discourse of ‘transition’ which positions Poland (and other postsocialist countries) as lagging behind the capitalist West. In this period postsocialist Poles again internalised the orientalist gaze which depicted the country as ‘backward’. Much like the partition periods, Polish intellectual elites identified societal groups ‘responsible’ for the failure of the national state after 1989. Some people were marked as ‘domestic others’ or ‘losers of transformation’ – those who are automatically proved to fail to adapt and to be ‘civilisationally incompetent’ or are unable to reject old mental habits, the *homo sovieticus* complex, who do not fit into this new civilised, post-communist reality of capitalism and progress (Buchowski, 2006). This internal orientalisation justifies a para-colonial relationship with western societies, casting them as more modern and representing a future which Poles aspire to, of underdeveloped ‘East’ and the civilised ‘West’ (Kania, 2009).

Domański (2004) suggests that the ideology of ‘catching up’ reinforces the acceptance of external influences, and a sense of exclusion from European integration after the Second World War leads to the acceptance of the recipient role and in turn to the reproduction of the East–West division. While assessing the Polish role in the European Union respondents in our research in Warsaw were appreciative of the financial benefits that accession to the EU brought, the active role that European institutions take in Polish domestic policies was rarely mentioned. Assessing Polish accession to the EU, respondents focused on differences in the standard of living that exist between Poland and Western Europe. The West was not only represented as better in terms of labour market opportunities and conditions, but also as ‘more developed’ in terms of social care and welfare, despite the fact that Poland was a ‘socialist welfare state’ in the past (Golinowska, 1994). Barbara, who has been the primary full-time carer for her disabled husband for more than 10 years, suggested that Western European countries represent a comfortable life to which she aspires:

So, those centres [for disabled] were founded, right, this is thanks to the European Union probably, but we are far behind, when it comes to any social assistance, suppose. I also had an uncle in West Germany, (…) [and] his wife died, he was left alone and he was also after the stroke, he immediately had such care as it should be, they brought him absolutely, completely out of it. Later (…) he had home care, a young woman did everything there until he died, right. Here [in Poland] there is no such assistance. (…) Social welfare in the West is more developed. (Barbara, 62)

The internalisation of the narrative of modernity is clear in this quote. Though the arrival of capitalism brought an end to many social provisions in Poland, it is not the Soviet Union that was ‘ahead’, but Western Europe. While state social care is being eroded in Western European countries as neoliberal capitalist economic agendas have risen to prominence (and in that sense the West is behind itself in terms of social welfare), what provision there is, nevertheless presents a future with which Poland shall one day ‘catch up’. Interestingly, here Germany is included in the western narrative not in the post-partition and post-war narrative, along with Russia. For example, some anti-EU debates regarding the possibility of buying land in Poland by foreigners were anchored in anti-German sentiments (Buchowski, 2010). This further exhibits the fluidity of discourses of the past and present with regard to relations with western hegemons.

Attitudes towards western countries are, indeed, a mix of desire and resentment, a negotiation, an ambivalent hybrid (Bhabha, 2005 [1994]). Western countries receive the highest scores in public opinion polls on perceptions of other nations, and have done for many years (CBOS, 2013). For decades Western Europe and the United States have constituted migration destinations for economic migrants from Poland and have been popularly depicted as ‘promised lands’ of prosperity. At the same time, Poles acknowledge that they are not always seen as desirable citizens in the West, and they have developed ‘a complex of the unwanted child’ (Horolets and Kozłowska, 2012: 51). Being ‘unwanted’ results in a sense of an uneasiness about one’s own position among other European countries, being not European enough, which is overcome by attempts to prove Polish superiority over the West in other dimensions. Poland is therefore often depicted in political and media debates as morally superior to western countries and as a society that has not been ‘spoiled’ by changes brought about by ‘civilisational’ processes (Wise, 2010b), for example its ethnic and religious homogeneity was valued by some. Reflecting on the multicultural projects pursued by some of the western countries respondents expressed scepticism in relation to the results in the UK, France, the Netherlands and Denmark. Beata, who has resided in a number of Western European countries, connected her Islamophobic feelings with this kind of argument:

I would very much not like for Poland to find itself in a situation like it is in France. At some point there was untamed immigration there. They had to accept people from the Maghreb, because it was their colony, and suddenly it turned out that those people were unwilling to integrate with society. They started living with their own enclaves, speak only in Arabic, and France started having whole Arabic cities. They started evicting the French from their estates because with time, more of them immigrated there and the value of those flats was lower, right? (…) Based on my observation of French, British and Dutch society, it seems that mass acceptance of migrants from Arabic countries has a negative impact on society in the long run.

What kind of risks are you talking about? (…)

The risk is of those people not wanting to accept the culture they are entering. (…) They don’t want to accept that value, those European values, they don’t want to accept human dignity, right? That man and woman have the same dignity and the same rights. They start living in their enclaves, I’m talking about the Netherlands, for example, right? They don’t learn the language, they act on their own law, they listen to their Imam more than, you know, the police or what the Dutch have to say. And for example I’d be against Warsaw, the city of Warsaw issuing a permit to build a mosque. (Beata, 37)

Here, racialised attitudes towards Muslim people intersect with a vision of the West, demonstrating how race is temporarily and spatially reconstructed (Meer and Nayak, 2013). As such, the applied triple relation anchored in postcolonial theory reveals the complexity of racialisation processes in a society that imagines its future through the experiences of the ‘civilised West’, to which it aspires, but also distances itself from. Approaching ethnic diversity from the perspective of an outsider (‘they have a problem’, Weinar, 2008: 5), despite being an insider in the EU, reinforces the self-representation of Poland as peripheral in Europe.

## Discussion

This article has presented original empirical material from research investigating the contemporary responses of Poles to ethnic diversity. We have here employed a postcolonial perspective with reference to ideas of Polishness and ‘otherness’, but, as we argue, these ordinary experiences have to be anchored in a *long-durée* perspective penetrating complex Polish history and hegemonic relations with other nations – either as a colonised or colonising power. The application of these postcolonial lenses has demonstrated that attitudes towards other nationalities are not merely a result of the ‘East–West split’ (Galbraith, 2004) or a by-product of the ‘postsocialist condition’ (Stenning, 2005). Rather, we propose that the contemporary Polish condition be considered in terms of the triple relation: in relation to Russia as its former colony reflecting past Russian Empire and Soviet domination, as a former coloniser of other Eastern European nations and in relation to the western hegemons.

The triple relation set out in the article provides a novel framework for understanding Polish identity within the context of three key external influences, drawing upon some of the central tropes of postcolonial theory. In doing so, it was not the aim of the article to provide a theory of prejudice within Poland. For instance, anti-Semitism, which continues to be present in Poland, could not be fully explored (see Cała, 2012). It is worth noting, however, that while postcolonial theory has not adequately addressed the issue of anti-Semitism, we would in this context distinguish between internal and external others. In the Polish case, Jews were (before the Holocaust) the ‘other’ within, and remain an ‘imagined internal other’ today. This prejudice is aligned with ideas of racial hierarchy in that being Jewish is related to descent, and is thus biologically unavoidable. This connects to popular ideas of a homogenous, mono-ethnic nation, which were pursued as an official policy of the state in the post-war period. Hierarchical conceptions of humanity and racial difference increased in Europe with the rise of the major colonial empires, and it was on this epistemological basis that anti-Semitism took hold in Germany and beyond. Racism and racialisation, including anti-Semitism, are therefore woven through all elements of the triple relation. As such, postcolonialism raises questions about dominant epistemologies which have long framed the world in hierarchical terms. Situating the analysis in racially homogenous Polish society paradoxically demonstrates ‘the resilience of race as a construct for organising social relations’ and how this ‘algebra of race’ is reconfigured across time and space selectively drawing upon past, present and imagined future (Meer and Nayak, 2013: 13). Through the postcolonial epistemological optic racial hierarchies, which include ‘invisible’ and ‘internal’ others such as Jews, other Eastern European nations and the working classes, go hand in hand with hierarchical ideas of civilisation.

Furthermore, the triple relation exposes constraints regarding the concept of modernity in its temporal and geo-political dimensions. While modernity is usually assumed to be a distinctive feature of western societies, the analysis provided demonstrates that Polish people make sense of their contemporary encounters with ethnic diversity by relating it to non-linear representations of past, present and future. This collective imaginary is relational and fluid and there is not one vision of modernity and change; for example Germany shifted from a category of colonisers to the western hegemons and Jewish people, as explained above, from ‘internal’ to ‘imagined’ others. As such, the data presented in this article have clearly demonstrated the ways in which ordinary people draw on aspects of this triple relation in making sense of both Polishness and ‘otherness’ in contemporary Poland. While more work is needed to develop this line of enquiry, we propose that concepts which have been developed in postcolonial studies have much to offer in terms of conceptualising and theorising processes of identity formation in the ‘power margins’ in Europe. Indeed, the complexities of national identities cannot be explained solely by historical events, but also the ways in which those events are subsumed into an ideological representation of past, present and future. What a postcolonial lens might offer, then, is an understanding of Polish national identity as mediated through a vision(s) of modernity: the modern society, the modern citizen, modern policies – what is especially important in the context of increased intra-EU mobility and Europeanisation of national politics. While Western Europe looms large in this framing of the world as a vision of the (or a possible) future, the triple relation draws attention to other relations also framed in terms of modernity which should be considered.
